# Natural progression of cardiac features and long-term effects of enzyme replacement therapy in Taiwanese patients with mucopolysaccharidosis II

**DOI:** 10.1186/s13023-021-01743-2

**Published:** 2021-02-23

**Authors:** Hsiang-Yu Lin, Ming-Ren Chen, Chung-Lin Lee, Shan-Miao Lin, Chung-Lieh Hung, Dau-Ming Niu, Tung-Ming Chang, Chih-Kuang Chuang, Shuan-Pei Lin

**Affiliations:** 1 Department of Medicine, MacKay Medical College, New Taipei City, Taiwan; 2 Department of Pediatrics, MacKay Memorial Hospital, No.92, Sec. 2, Chung-Shan North Road, Taipei, 10449 Taiwan; 3 Department of Medical Research, MacKay Memorial Hospital, 92 Chung-Shan N. Rd., Sec. 2, Taipei, 10449 Taiwan; 4 Department of Childhood Care and Education, MacKay Junior College of Medicine, Nursing and Management, Taipei, Taiwan; 5 Department of Medical Research, China Medical University Hospital, China Medical University, Taichung, Taiwan; 6 Department of Rare Disease Center, MacKay Memorial Hospital, Taipei, Taiwan; 7 Department of Pediatrics, MacKay Memorial Hospital, Hsinchu, Taiwan; 8 Institute of Clinical Medicine, National Yang-Ming University, Taipei, Taiwan; 9 Division of Cardiology, Department of Internal Medicine, MacKay Memorial Hospital, Taipei, Taiwan; 10 Department of Pediatrics, Taipei Veterans General Hospital, Taipei, Taiwan; 11 Department of Pediatric Neurology, Changhua Christian Children’s Hospital, Changhua, Taiwan; 12 School of Medicine, Kaohsiung Medical University, Kaohsiung, Taiwan; 13 College of Medicine, Fu-Jen Catholic University, Taipei, Taiwan; 14 Department of Infant and Child Care, National Taipei University of Nursing and Health Sciences, Taipei, Taiwan

**Keywords:** Cardiac hypertrophy, Echocardiography, Mucopolysaccharidosis II, Valvular heart disease

## Abstract

**Background:**

Cardiac abnormalities have been observed in patients with mucopolysaccharidosis type II (MPS II). The aim of this study was to investigate the cardiac features and natural progression of Taiwanese patients with MPS II, and evaluate the impact of enzyme replacement therapy (ERT) on cardiac structure and function.

**Methods:**

The medical records and echocardiograms of 48 Taiwanese patients with MPS II (median age, 6.9 years; age range, 0.1–27.9 years) were reviewed. The relationships between age and each echocardiographic parameter were analyzed.

**Results:**

The mean *z*-scores of left ventricular mass index (LVMI), interventricular septum diameter in diastole (IVSd), left ventricular posterior wall diameter in diastole (LVPWd), and aortic diameter were 1.10, 2.70, 0.95 and 1.91, respectively. *Z* scores > 2 were identified in 33%, 54%, 13%, and 46% for LVMI, IVSd, LVPWd, and aortic diameter, respectively. The most prevalent cardiac valve abnormality was mitral regurgitation (MR) (56%), followed by aortic regurgitation (AR) (33%). The severity of mitral stenosis (MS), MR, aortic stenosis (AS), AR, and the existence of valvular heart disease were all positively correlated with increasing age (*p* < 0.01). We also compared the echocardiographic parameters between two groups: (1) 12 patients who had up to 17 years of follow-up echocardiographic data without ERT, and (2) nine patients who had up to 12 years of follow-up data with ERT. The results showed that *z*-score changes of LVMI significantly improved in the patients who received ERT compared to those who did not receive ERT (0.05 versus 1.52, *p* < 0.05). However, the severity score changes of MS, MR, AS, and AR all showed gradual progression in both groups (*p* > 0.05).

**Conclusions:**

High prevalence rates of valvular heart disease and cardiac hypertrophy were observed in the MPS II patients in this study. The existence and severity of cardiac hypertrophy and valvular heart disease in these patients worsened with increasing age, reinforcing the concept of the progressive nature of this disease. ERT for MPS II appeared to be effective in stabilizing or reducing the progression of cardiac hypertrophy, but it only had a limited effect on valvulopathy.

## Introduction

Mucopolysaccharidoses (MPSs; OMIM 252700) are a group of lysosomal storage diseases caused by deficiencies in specific lysosomal enzymes that involve the sequential catabolism of glycosaminoglycans (GAGs) leading to progressive substrate accumulation in various tissues and organs. Seven different types of MPS disorders (I, II, III, IV, VI, VII, and IX) with 11 specific lysosomal enzyme deficiencies have been reported. MPS has a variable age at onset of symptoms and variable rate of progression [1]. MPS II (Hunter syndrome; OMIM +309900) is an inherited X-linked recessive disease caused by deficient activity of iduronate-2-sulfatase (IDS), which catalyzes a sequential step in the degradation of the GAGs dermatan sulfate and heparan sulfate. Patients with the severe form of MPS II usually manifest between 2 and 4 years of age with coarse face, recurrent ear, nose, and throat infections, airway obstruction, cardiac valve dysplasia, cardiomyopathy, hepatosplenomegaly, hernias, skeletal deformities, joint stiffness, and progressive neurological deterioration, leading to profound cognitive impairment. Patients with the mild form of MPS II still have miscellaneous somatic problems, but without cognitive impairment [1, 2]. MPS II is one of the most common MPSs, with an approximate prevalence of 1 in 140,000–156,000 live births in Europe [3]. In Asian countries, the incidence of MPS II is higher than the other types of MPSs [4]. In Taiwan, the incidence of MPS II is estimated to be 1 in 94,000 live births [5].

Cardiac valve thickening, valvular regurgitation and stenosis, cardiac hypertrophy, and aortic root dilatation are common cardiovascular defects of MPS II [6–18]. GAG accumulation in the cardiac valves, myocardium, great vessels, and coronary arteries leads to valvular defects and cardiomyopathy [19]. Valvular stenosis and regurgitation caused by mitral or aortic leaflet thickening, calcification, and cardiac dysfunction resulting from deformities in cardiac structures are associated with significant increases in morbidity and mortality. Heart failure, sudden death from arrhythmias, and coronary occlusion are all cardiac causes of death [20–23].

Enzyme replacement therapy (ERT) with recombinant IDS (idursulfase; Elaprase, Shire Human Genetic Therapies, Cambridge, MA, USA) has been licensed in the United States and the European Union for the treatment of MPS II for over a decade. ERT has been demonstrated to be beneficial for many patients with MPS II, with improvements in endurance, joint mobility, lung function, and quality of life, especially if started early in the course of the disease [24–28]. Previous studies have demonstrated that long-term ERT for MPS II reduces or at least stabilizes left ventricular mass index (LVMI) and interventricular septal hypertrophy, but it does not improve valvular regurgitation or stenosis [10, 13, 15, 26, 28–30]. Nonetheless, only a few reports have focused on the natural progression of cardiac alterations and long-term effects of ERT in MPS II patients in Asia. The purpose of this study was to investigate the cardiac features and natural progression of Taiwanese patients with MPS II, and evaluate the impact of ERT on cardiac structure and function.

## Materials and methods

### Study population

The medical records and echocardiograms of 48 Taiwanese patients with MPS II (mean age, 8.4 ± 6.5 years; median age, 6.9 years; age range, 0.1–27.9 years) were retrospectively reviewed at MacKay Memorial Hospital from December 1992 to February 2020. The diagnosis of all patients was confirmed by a deficiency of IDS activity measured in peripheral leukocytes or fibroblasts, two-dimensional electrophoresis of urinary GAGs, quantitative determination of dermatan sulfate and heparan sulfate using liquid chromatography-mass spectrometry, and/or mutational analysis [31–33]. None of the patients had received ERT or hematopoietic stem cell transplantation (HSCT) at baseline. We also reviewed and analyzed 12 patients with up to 17 years of follow-up echocardiographic data without ERT or HSCT, and nine patients with up to 12 years of follow-up data who had received ERT with 0.5 mg/kg/week intravenous idursulfase (Elaprase). Written informed consent for cardiac evaluations was obtained from a parent for children and from the patients if they were over 18 years of age. This study was approved by the Ethics Committee of MacKay Memorial Hospital, Taipei, Taiwan.

### Measurements of echocardiographic parameters

A Philips Sonos 5500/7500 ultrasound system (Andover, MA, USA) equipped with electronic transducers from 2 to 8 MHz was used to measure echocardiographic parameters. One experienced cardiologist (MRC) digitally stored and analyzed the data to minimize inter-observer variations. Left ventricular (LV) systolic and diastolic diameters were measured in M-mode. LV systolic function was assessed on the basis of the ejection fraction in accordance with the Simpson method. For children, an ejection fraction of < 50% was defined as being abnormal, while for adults, an ejection fraction of < 52% for men and < 54% for women were defined as being abnormal [34]. Asymmetric septal hypertrophy was considered to be present if the LV interventricular septum/posterior wall thickness ratio in end-diastole was ≥ 1.5 [35]. Diastolic filling was evaluated according to the E/A ratio by measuring mitral-inflow using pattern-peak early filling (E) and late filling (A) velocities [36]. The presence of diastolic dysfunction was indicated by a reversed E/A ratio (E/A ratio < 1). The severity of valvular stenosis and regurgitation was evaluated and graded as follows: 0 (none), 1 (mild), 2 (moderate), and 3 (severe), according to the European Society of Cardiology guidelines [37–40]: mild aortic stenosis (AS) = a valve area > 1.5 cm^2^ and a mean gradient < 30 mmHg; moderate AS = a valve area of 1.0–1.5 cm^2^ and a mean gradient of 30–50 mmHg; severe AS = a valve area < 1.0 cm^2^ and a mean gradient > 50 mmHg; mild mitral stenosis (MS) = a valve area > 1.5 cm^2^ and a mean gradient < 5 mmHg; moderate MS = a valve area between 1.0–1.5 cm^2^ and a mean gradient between 5–10 mmHg; and severe MS = a valve area < 1.0 cm^2^ and a mean gradient > 10 mmHg. Because of the high frequency of physiological tricuspid regurgitation in the general population, we did not regard tricuspid regurgitation as a pathological finding in this study.

The echocardiographic data of LVMI, right ventricular end-diastolic dimension (RVDd), interventricular septal end-diastolic dimension (IVSd) and end-systolic, left ventricular end-diastolic dimension (LVIDd) and end-systolic (LVIDs), left ventricular posterior wall end-diastolic dimension (LVPWd) and end-systolic, aortic diameter, and left atrial dimension (LAD) were recorded. The relative wall thickness (RWT) was calculated as (2 × LVPWd)/LVIDd. Three patterns of LV remodeling were defined on the basis of assessments of the LVMI and RWT, including concentric remodeling (normal LVMI and RWT > 0.42), eccentric hypertrophy (LVMI *z*-score > 2 and RWT ≤ 0.42), and concentric hypertrophy (LVMI *z*-score > 2 and RWT > 0.42) [41]. The aorta was measured on the sinus from the leading edge to leading edge. LVMI was calculated using the Devereux formula and indexed by the height *z*-score with normal values according to the report by Foster et al. [42]. These results were compared with normal values based on the study by Kampmann et al. [43].

We transformed all echocardiographic parameters into a *z*-score derived by subtracting the mean reference value from an individual observed value, then dividing the difference by the standard deviation from the reference value. A *z*-score value between − 2 and + 2 was considered to be normal.

### Data analysis and statistics

Sex, age, height, weight, and body surface area at the time of echocardiographic evaluations were recorded for each patient. Descriptive statistics including means and standard deviations of all echocardiographic parameters were calculated. Relationships between age and different echocardiographic parameters were analyzed using Pearson’s correlation coefficient (*r*), and significance was assessed using Fisher’s *r–z* transformations. We compared changes in *z*-scores of LVMI, IVSd, and LVPWd, and severity scores of MS, MR, AS, and AR for 12 patients without ERT compared to nine patients with ERT. Two-tailed *p* values were computed. All statistical analyses were performed using SPSS version 11.5 (SPSS Inc., Chicago, Illinois, USA). Differences with *p* < 0.05 were considered to be statistically significant.

## Results

**Fig. 1:**
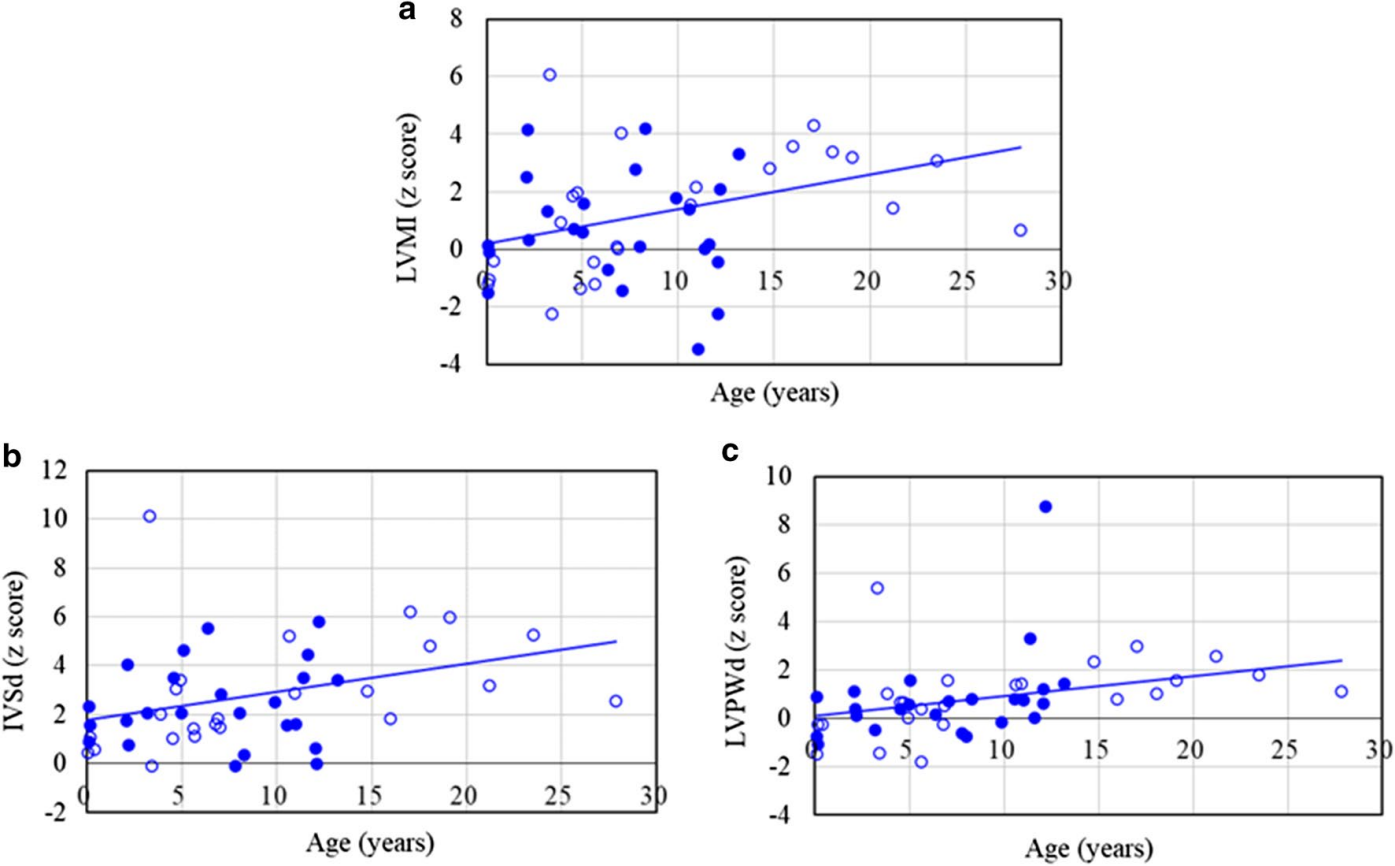
The relationships between age and *z-*scores of **a** LVMI (*r* = 0.300, *p* < 0.05), **b** IVSd (*r* = 0.325, *p* < 0.05), and **c** LVPWd (*r* = 0.379, *p* < 0.01) in 48 patients with MPS II. The line represents the trendline. The open and closed circles represent mild and severe forms of patients with MPS II, respectively. LVMI, left ventricular mass index; IVSd, interventricular septum thickness in diastole; LVPWd, left ventricular posterior wall thickness in diastole

**Fig. 2:**
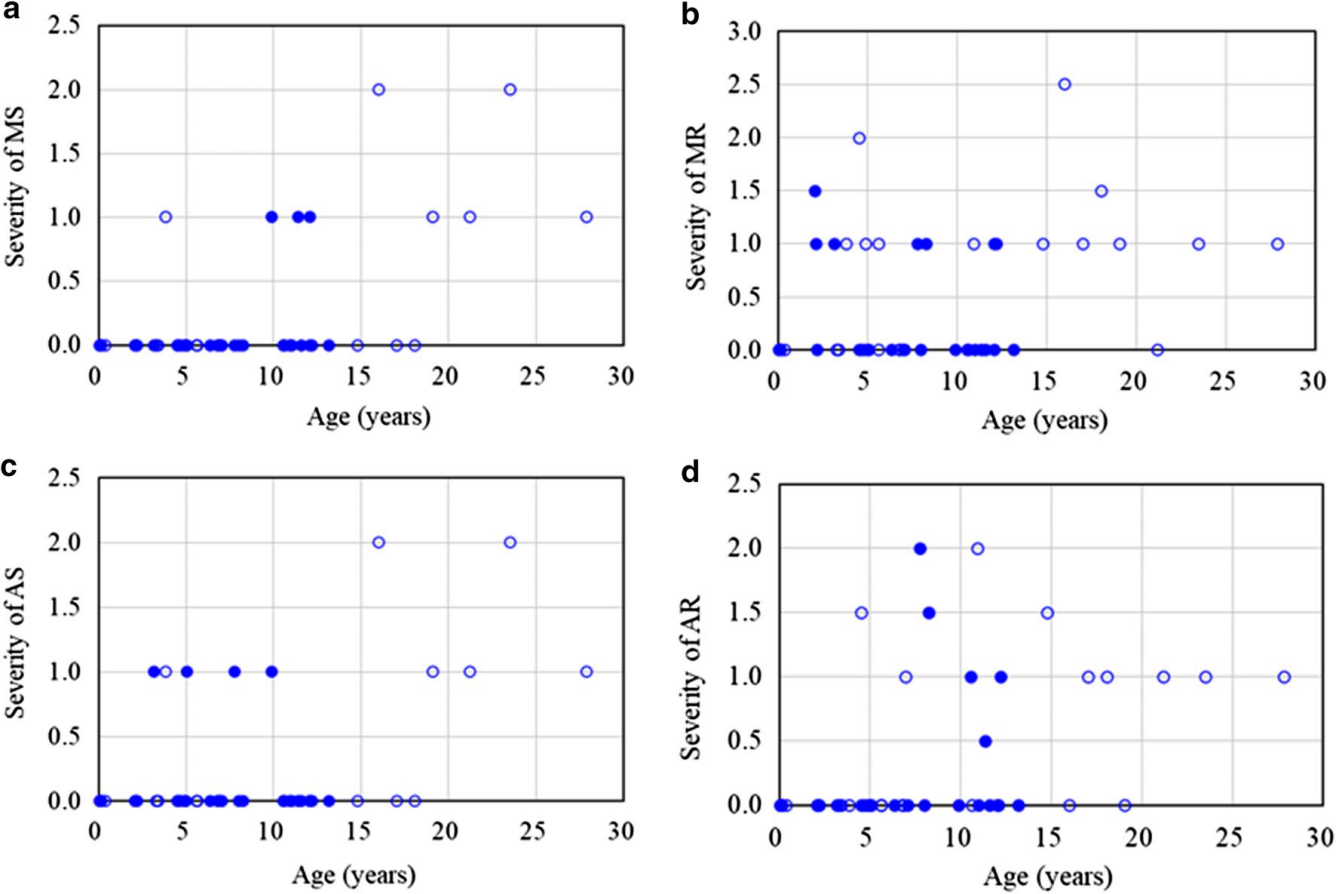
The relationships between age and severity of cardiac valve abnormalities in the 48 patients with MPS II (severity score: 3: severe, 2: moderate, 1: mild, 0: normal). **a** MS, mitral stenosis (*r* = 0.629, *p* < 0.01); **b** MR, mitral regurgitation (*r* = 0.386, *p* < 0.01); **c** AS, aortic stenosis (*r* = 0.510, *p* < 0.01); **d** AR, aortic regurgitation (*r* = 0.477, *p* < 0.01). The blue circles represent the age and severity of cardiac valve abnormalities in the 48 patients. The open and closed circles represent mild and severe forms of patients with MPS II, respectively

Tables 1 and 2 show the baseline clinical, echocardiographic and mutational studies of the 48 patients with MPS II. There were 24 neuronopathic and 24 non-neuronopathic patients in this study. Most of these patients (n = 41, 85%) had cardiac abnormalities, and only seven who were referred from newborn screening programs (Patients No. 1, 2, 4–6, 12, 23) had normal cardiac features by echocardiography. The mean *z*-scores of LVMI, IVSd, LVPWd, and aortic diameter were 1.05, 2.66, 0.86 and 1.97, respectively. *Z*-scores > 2 were identified in 31%, 54%, 13%, and 46% for LVMI, IVSd, LVPWd, and aortic diameter, respectively. *Z*-scores for LVMI, IVSd and LVPWd were all positively correlated with increasing age (*p* < 0.05) (Table 3 and Fig. 1). Echocardiographic examinations (n = 48) revealed that 37 patients (77%) had valvular heart disease, 17 (35%) had valvular stenosis, and 31 (65%) had regurgitation. The most prevalent cardiac valve abnormality was mitral regurgitation (MR) (54%), followed by aortic regurgitation (AR) (35%). Thirteen (27%), 22 (46%) and four (8%) patients had mitral valve prolapse, a thickened IVS, and asymmetric septal hypertrophy, respectively. The severity of MS, MR, AS, and AR, and the existence of valvular heart disease, LV remodeling pattern abnormality, and thick IVS were all positively correlated with increasing age (*p* < 0.01). There is no difference in cardiac findings between severe and mild forms of MPS II (Fig. 2). Diastolic dysfunction [reversed ratio between early and late (atrial) ventricular filling velocity (E/A ratio < 1)] was identified in eight patients (17%), however, systolic dysfunction (abnormal ejection fraction) was found in only one patient (2%) (Table 1). Ten (21%), four (8%), and three (6%) patients had LV eccentric hypertrophy, concentric remodeling, and concentric hypertrophy, and this was associated with a higher risk of subsequent cardiovascular events compared to the other 31 patients (65%) with normal LV geometry. Additional file 1: Tables 1 and 2 show the detailed data of 12 patients with MPS II who had echocardiographic examinations after 2.6–17.0 years of follow-up and had not received ERT or HSCT. Compared to baseline, the mean *z*-score increases were 1.52, 1.80, and 1.25 for LVMI, IVSd, and LVPWd, respectively, indicating the natural progression of hypertrophic cardiomyopathy. The severity score increases were 0.42, 0.54, 0.54, and 0.75 for MS, MR, AS, and AR, respectively, indicating the natural deterioration of valvulopathy. Additional file 1: Tables 3 and 4 show the baseline and follow-up echocardiographic parameters of nine patients with MPS II who received ERT for 1.0–12.4 years. The mean *z*-score changes were 0.05, -0.24, and 0.52 for LVMI, IVSd, and LVPWd, respectively. This showed that ERT stabilized or alleviated the natural progression of hypertrophic cardiomyopathy. In addition, the *z*-score changes of LVMI showed greater improvements in patients No. 25, 33, and 20, all of whom started receiving ERT at a relatively younger age. The *z*-score changes of LVMI in these three patients were -0.64, -0.58, and -1.21, respectively (Additional file 1: Table 3). The *z*-score changes of LVMI showed significant improvements in the patients who received ERT compared to those who did not receive ERT (0.05 versus 1.52, *p* < 0.05) (Table 4). However, the severity scores of MS, MR, AS, and AR all revealed gradual progression in both the patients with and without ERT (*p* > 0.05), indicating the limited effects of ERT for valvular heart diseases (Table 5).

**Table 1:** Baseline clinical and echocardiographic features of the 48 patients with MPS II

No	Gender	MPS type	Age (years)	LVMI (z score)	RVDd (z score)	IVSd (z score)	IVSs (z score)	LVIDd (z score)	LVIDs (z score)	LVPWd (z score)	LVPWs (z score)	AoD (z score)	LAD (z score)	EF (%)	Reversed E/A ratio
1	M	II (M)	0.1	− 1.21	− 0.05	0.46	− 0.01	1.11	0.87	− 1.48	− 0.51	0.50	0.00	66	−
2	M	II (S)	0.1	− 1.52	0.07	0.90	− 0.23	− 1.28	− 0.13	− 0.78	− 1.42	− 0.33	− 2.29	55	−
3	M	II (S)	0.1	0.13	0.89	**2.34**	0.18	0.67	1.27	0.86	− 0.05	0.50	− 1.71	58	−
4	M	II (M)	0.1	− 1.05	0.11	1.07	− 0.05	− 1.02	− 0.05	− 0.25	− 1.57	− 0.26	− 1.21	57	−
5	M	II (S)	0.2	− 0.09	− 0.68	1.59	− 1.33	0.94	1.75	− 1.08	− 0.54	− 0.26	− 1.21	56	−
6	M	II (M)	0.4	− 0.42	0.15	0.57	− 0.56	0.65	1.35	− 0.25	− 1.28	1.00	− 1.53	60	−
7	M	II (S)	2.1	**2.49**	**2.09**	1.73	0.72	**2.40**	**2.68**	1.09	**2.42**	**3.20**	1.19	63	−
8	M	II (S)	2.2	**4.16**	− 0.38	**4.04**	0.21	**5.48**	**6.65**	0.37	− 0.77	**3.92**	**2.59**	52	−
9	M	II (S)	2.2	0.33	− 0.24	0.73	1.08	0.83	0.54	0.11	0.48	**4.18**	1.12	69	−
10	M	II (S)	3.2	1.30	1.73	**2.07**	0.91	1.91	0.73	− 0.48	1.23	1.82	− 1.28	74	−
11	M	II (M)	3.3	**6.06**	0.27	**10.13**	**3.27**	1.20	0.42	**5.40**	**3.00**	**2.94**	0.84	72	−
12	M	II (M)	3.4	− 2.26	0.50	− 0.12	− 0.12	0.07	1.32	− 1.45	− 1.04	0.53	− 0.34	58	−
13	M	II (M)	3.8	0.95	1.32	2.00	0.58	0.68	0.42	1.00	0.55	0.06	− 0.88	69	−
14	M	II (M)	4.5	1.87	0.32	1.01	− 0.28	**2.92**	**2.38**	0.66	0.35	2.00	0.08	67	−
15	M	II (S)	4.5	0.71	1.06	**3.51**	1.56	− 0.84	− 1.44	0.38	0.57	0.88	0.37	75	−
16	M	II (M)	4.7	1.96	1.32	**3.04**	1.45	1.03	1.44	0.63	− 1.01	**3.71**	− 1.43	62	−
17	M	II (M)	4.9	− 1.38	0.04	**3.41**	1.33	− 3.10	− 2.64	0.00	0.03	0.06	− 3.17	71	−
18	M	II (S)	5.0	0.60	1.09	**2.07**	0.05	− 0.85	− 1.61	0.57	**2.67**	1.28	− 0.71	77	−
19	M	II (S)	5.1	1.57	**2.39**	**4.61**	1.12	− 1.74	− 1.93	1.57	**2.07**	0.67	− 0.10	75	−
20	M	II (M)	5.6	− 0.43	0.38	1.45	0.63	1.29	1.76	− 1.80	− 1.60	0.71	− 0.30	61	−
21	M	II (M)	5.6	− 1.21	**2.60**	1.13	0.63	− 1.16	− 0.96	0.38	1.45	0.24	− 1.17	68	−
22	M	II (S)	6.4	− 0.71	**2.18**	**5.53**	**2.60**	− 2.09	− 1.46	0.16	− 0.24	**3.29**	− 1.32	67	+
23	M	II (M)	6.8	0.07	1.96	1.59	0.41	0.20	0.82	− 0.25	− 0.99	0.72	− 1.35	60	−
24	M	II (M)	6.9	0.03	0.87	1.83	0.28	− 1.41	− 1.36	0.53	0.31	1.89	− 1.45	71	−
25	M	II (M)	7.0	**4.03**	− 0.85	1.47	**2.30**	**3.47**	**2.62**	1.57	1.63	**2.50**	− 1.62	66	−
26	M	II (S)	7.1	− 1.46	NA	**2.83**	− 0.03	− 3.58	− 2.96	0.70	− 0.20	**4.65**	− 3.40	71	−
27	M	II (S)	7.8	**2.78**	− 1.32	− 0.11	− 0.35	**5.97**	**6.68**	− 0.62	0.52	**4.61**	− 1.35	**46**	−
28	M	II (S)	8.0	0.10	− 0.93	**2.06**	1.18	0.36	− 0.43	− 0.77	− 0.13	**2.06**	− 0.26	73	−
29	M	II (S)	8.3	**4.19**	− 1.28	0.37	− 0.65	**7.15**	**6.86**	0.80	0.19	**3.28**	0.03	53	−
30	M	II (S)	9.9	1.77	0.94	**2.50**	1.45	0.74	− 0.28	− 0.18	0.76	1.94	0.83	74	+
31	M	II (S)	10.6	1.41	1.33	1.56	0.30	0.03	− 0.83	0.80	1.41	1.17	− 1.45	75	−
32	M	II (M)	10.6	1.55	0.59	**5.20**	2.00	0.12	0.46	1.36	− 0.50	**2.65**	1.04	65	−
33	M	II (M)	10.9	**2.16**	− 0.21	**2.88**	1.70	1.97	1.24	1.42	1.63	**2.94**	− 2.21	68	−
34	M	II (S)	11.0	− 3.48	0.26	1.60	− 0.66	− 3.19	− 2.08	0.74	− 0.23	**2.47**	− 2.80	63	+
35	M	II (S)	11.4	0.00	0.59	**3.51**	**2.07**	− 1.63	− 1.88	**3.31**	**2.08**	**3.06**	− 2.52	75	−
36	M	II (S)	11.6	0.16	NA	**4.46**	**2.77**	− 0.61	− 1.32	0.02	0.83	**2.71**	0.37	75	−
37	M	II (S)	12.1	− 0.44	1.00	0.62	− 0.21	− 1.24	− 0.97	1.18	1.78	1.44	− 1.90	68	−
38	M	II (S)	12.1	− 2.25	− 0.05	0.00	**2.07**	− 0.89	− 3.96	0.59	**3.31**	**2.76**	0.52	93	−
39	M	II (S)	12.2	**2.07**	NA	**5.79**	**5.03**	− 3.35	− 3.92	**8.76**	**5.38**	1.24	0.87	83	−
40	M	II (S)	13.2	**3.32**	1.54	**3.40**	**2.24**	0.21	− 0.69	1.42	1.26	**3.17**	− 1.24	75	+
41	M	II (M)	14.8	**2.81**	− 1.35	**2.94**	0.37	1.65	0.86	**2.35**	− 1.33	**2.51**	− 1.22	67	−
42	M	II (M)	16.0	**3.57**	NA	1.84	0.25	**4.82**	**4.69**	0.80	− 0.01	1.78	**4.24**	58	−
43	M	II (M)	17.0	**4.32**	NA	**6.22**	**4.12**	1.02	− 0.43	**2.95**	**3.48**	**4.28**	− 0.58	76	−
44	M	II (M)	18.1	**3.37**	− 1.93	**4.82**	**2.81**	**3.28**	0.62	1.04	**2.17**	**4.07**	1.19	76	−
45	M	II (M)	19.1	**3.19**	0.57	**6.00**	**3.12**	− 0.07	− 1.00	1.57	1.78	**3.06**	0.58	76	+
46	M	II (M)	21.2	1.44	NA	**3.18**	**2.67**	− 2.42	− 3.18	**2.55**	**2.32**	1.54	0.59	79	+
47	M	II (M)	23.5	**3.09**	NA	**5.27**	2.00	0.13	− 0.89	1.79	1.26	0.85	− 1.61	75	+
48	M	II (M)	27.9	0.65	**5.52**	**2.56**	− 0.06	− 0.82	− 1.00	1.08	− 1.04	0.67	0.21	70	+

MPS, mucopolysaccharidosis; LVMI, left ventricular mass index; RVDd, right ventricular end-diastolic dimension; IVSd, interventricular septal end-diastolic dimension; IVSs, interventricular septal end-systolic dimension; LVIDd, left ventricular end-diastolic dimension; LVIDs, left ventricular end-systolic dimension; LVPWd, left ventricular posterior wall end-diastolic dimension; LVPWs, left ventricular posterior wall end-systolic dimension; AoD, aortic diameter; LAD: left atrial dimension; EF, ejection fraction; E/A: ratio between early and late (atrial) ventricular filling velocity; MPS II (S), severe form; MPS II (M), mild form; NA, not available. The values of z score > 2 are presented in boldface

**Table 2:** Baseline clinical, echocardiographic and mutational studies of the 48 patients with MPS II

No	Gender	MPS type	Age (years)	MS	MR	AS	AR	MVP	Thick IVS	ASH	Left ventricular remodeling pattern	*IDS* gene mutation
1	M	II (M)	0.1	0	0	0	0	−	−	−	Normal geometry	c.817C>T; p.R273W
2	M	II (S)	0.1	0	0	0	0	−	−	−	Normal geometry	c.311 A>T; p.D104V
3	M	II (S)	0.1	0	0	0	0	−	−	−	Normal geometry	c.1007-1666_c.1180 + 2113 delinsTT (including exon 8 del)
4	M	II (M)	0.1	0	0	0	0	−	−	−	Normal geometry	c.1025A>G; p.H342R
5	M	II (S)	0.2	0	0	0	0	−	−	−	Normal geometry	c.1400C>T; p.P467L
6	M	II (M)	0.4	0	0	0	0	−	−	−	Normal geometry	c.254C>T; p.A85V
7	M	II (S)	2.1	0	1.5	0	0	+	−	−	Eccentric hypertrophy	exon 2 del121-123
8	M	II (S)	2.2	0	1	0	0	+	−	+	Eccentric hypertrophy	c.240+1G>C
9	M	II (S)	2.2	0	0	0	0	−	−	−	Normal geometry	IDS inversion
10	M	II (S)	3.2	0	1	1	0	+	−	−	Normal geometry	*IDS* intron 7 to IDS2 intron 1, 254,436 to 294,456 (recombination)
11	M	II (M)	3.3	0	2	0	0	+	−	−	Concentric hypertrophy	NA
12	M	II (M)	3.4	0	0	0	0	−	−	−	Normal geometry	c.1025A>G; p.H342R
13	M	II (M)	3.8	1	1	1	0	−	+	−	Normal geometry	c.1122C>T; p.Gly374=
14	M	II (M)	4.5	0	2	0	1.5	−	+	−	Normal geometry	c.683C>T; p.P228L
15	M	II (S)	4.5	0	0	0	0	+	+	−	Normal geometry	Exon 4–7 deletion
16	M	II (M)	4.7	0	0	0	0	−	−	−	Normal geometry	NA
17	M	II (M)	4.9	0	1	0	0	−	+	−	Concentric remodeling	c.507+1G>A
18	M	II (S)	5.0	0	1	0	0	+	+	−	Normal geometry	c.262C>T; p.R88C
19	M	II (S)	5.1	1	0	1	0	−	+	−	Concentric remodeling	NA
20	M	II (M)	5.6	0	1	0	0	−	−	+	Normal geometry	c.253G>A; p.A85T
21	M	II (M)	5.6	0	0	0	0	−	−	−	Normal geometry	IVS7+5G>C (22 bp ins)
22	M	II (S)	6.4	1	1	0	0	−	+	+	Normal geometry	NA
23	M	II (M)	6.8	0	0	0	0	−	−	−	Normal geometry	c.817C>T; p.R273W
24	M	II (M)	6.9	0	0	0	0	+	+	−	Normal geometry	c.1403G>A; p.R468Q
25	M	II (M)	7.0	0	0	0	1	−	−	−	Eccentric hypertrophy	IDS inversion
26	M	II (S)	7.1	0	0	0	0	−	−	−	Normal geometry	c.1402C>T; p.R468W
27	M	II (S)	7.8	0	1	1	2	−	−	−	Normal geometry	c.454A>C; p.S152R
28	M	II (S)	8.0	0	0	0	1.5	−	+	−	Normal geometry	c.231_236delCTTTGC
29	M	II (S)	8.3	0	1	0	1.5	+	−	−	Eccentric hypertrophy	c.1466G>A; p.G489D
30	M	II (S)	9.9	1	0	1	0	−	+	−	Normal geometry	c.262C>T; p.R88C
31	M	II (S)	10.6	0	1	0	1	−	−	−	Normal geometry	c.1402C>T; p.R468W
32	M	II (M)	10.6	1	0	1	0	−	+	−	Normal geometry	c.801G>T; p.W267C
33	M	II (M)	10.9	0	1	0	2	−	+	−	Eccentric hypertrophy	c.1006+5G>C
34	M	II (S)	11.0	1	1	0	0	+	−	−	Normal geometry	c.253G>A; p.A85T
35	M	II (S)	11.4	1	0	0	0.5	−	+	−	Normal geometry	NA
36	M	II (S)	11.6	1	1	1	1.5	+	+	+	Normal geometry	c.413A>G; p.H138R
37	M	II (S)	12.1	1	1	0	0	−	−	−	Normal geometry	c.1402C>T; p.R468W
38	M	II (S)	12.1	0	0	0	0.5	−	−	−	Normal geometry	c.801G>T; p.W267C
39	M	II (S)	12.2	0	1	0	1	−	+	−	Concentric remodeling	c.1454T>G; p.I485R
40	M	II (S)	13.2	1	1	1	0	−	+	−	Eccentric hypertrophy	c.1402C>T; p.R468W
41	M	II (M)	14.8	0	1	0	1.5	+	+	−	Eccentric hypertrophy	c.1006+5G>C
42	M	II (M)	16.0	2	2.5	2	0	−	−	−	Eccentric hypertrophy	c.697A>G; p.R233G
43	M	II (M)	17.0	0	1	0	1	+	−	−	Concentric hypertrophy	c.1122C>T; p.Gly374=
44	M	II (M)	18.1	0	1.5	0	1	+	+	−	Eccentric hypertrophy	c.1006+5G>C
45	M	II (M)	19.1	1	1	1	0	−	+	−	Eccentric hypertrophy	NA
46	M	II (M)	21.2	1	0	1	1	−	+	−	Concentric remodeling	c.1122C>T; p.Gly374=
47	M	II (M)	23.5	2	1	2	1	−	+	−	Concentric hypertrophy	c.801G>T; p.W267C
48	M	II (M)	27.9	1	1	1	1	−	+	−	Normal geometry	del exon 8

MPS, mucopolysaccharidosis; MS, mitral stenosis; MR, mitral regurgitation; AS, aortic stenosis; AR, aortic regurgitation; MVP, mitral valve prolapse; IVS, interventricular septum; ASH, asymmetric septal hypertrophy; MPS II (S), severe form; MPS II (M), mild form; NA, not available. Severity of valvular stenosis and regurgitation (MS, MR, AS, AR) were estimated and graded on the following scores: 0 (none), 1 (mild), 2 (moderate), and 3 (severe)

**Table 3:** The values of echocardiographic parameters of left ventricular chamber dimensions and function of the 48 patients with MPS II

Echocardiographic parameters	LVMI (*z* score)	RVDd (*z* score)	IVSd (*z* score)	IVSs (*z* score)	LVIDd (*z* score)	LVIDs (*z* score)	LVPWd (*z* score)	LVPWs (*z* score)	AoD (*z* score)	LAD (*z* score)
Mean (SD)	1.05 (2.05)	0.60 (1.33)	2.66 (2.04)	1.06 (1.36)	0.44 (2.35)	0.23 (2.44)	0.86 (1.73)	0.68 (1.53)	1.97 (1.41)	− 0.56 (1.46)
*r* value (z score versus age)	0.300	0.211	0.325	0.420	− 0.084	− 0.267	0.379	0.314	0.228	0.172
*p* value	*p* < 0.05	*p* > 0.05	*p* < 0.05	*p* < 0.01	*p* > 0.05	*p* > 0.05	*p* < 0.01	*p* > 0.05	*p* > 0.05	*p* > 0.05
*z* score > 2, n (%)	15 (31%)	5 (12%)	26 (54%)	12 (25%)	8 (17%)	7 (15%)	6 (13%)	10 (21%)	22 (46%)	2 (4%)

MPS, mucopolysaccharidosis; LVMI, left ventricular mass index; RVDd, right ventricular end diastolic dimension; IVSd, interventricular septum thickness in diastole; IVSs, interventricular septum thickness in systole; LVIDd, left ventricular internal diameter in diastole; LVIDs, left ventricular internal diameter in systole; LVPWd, left ventricular posterior wall thickness in diastole; LVPWs, left ventricular posterior wall thickness in systole; AoD, aortic diameter; LAD: left atrial dimension; SD, standard deviation

**Table 4:** The mean age at baseline and follow-up, and changes in z-scores of LVMI, IVSd, and LVPWd for 12 patients without ERT compared to nine patients with ERT

ERT		Age (years)	Duration (years)	LVMI (z score)	Change	IVSd (z score)	Change	LVPWd (z score)	Change
Without ERT (n = 12)	Baseline	7.3	8.1 (2.6–17.0)	0.83	1.52	2.40	1.80	0.58	1.25
	Follow-up	15.4		2.35		4.20		1.82	
With ERT (n = 9)	Baseline	15.2	7.2 (1.0–12.4)	2.78	0.05	3.01	-0.24	1.68	0.52
	Follow-up	22.4		2.83		2.77		2.19	
*p* value				*p* = 0.013		*p* = 0.069		*p* = 0.358	

MPS, mucopolysaccharidosis; ERT, enzyme replacement therapy; LVMI, left ventricular mass index; IVSd, interventricular septum thickness in diastole; LVPWd, left ventricular posterior wall thickness in diastole

**Table 5:** The mean age at baseline and follow-up, and changes in severity scores of MS, MR, AS, and AR for 12 patients without ERT compared to nine patients with ERT

ERT		Age (years)	Duration (years)	MS severity score	Change	MR severity score	Change	AS severity score	Change	AR severity score	Change
Without ERT (n = 12)	Baseline	7.3	8.1 (2.6–17.0)	0.33	0.42	0.63	0.54	0.25	0.54	0.13	0.75
	Follow-up	15.4		0.75		1.17		0.79		0.88	
With ERT (n = 9)	Baseline	15.2	7.2 (1.0–12.4)	0.44	0.44	0.78	0.06	0.44	0.67	1.44	0.39
	Follow-up	22.4		0.89		0.83		1.11		1.83	
*p* value				*p* = 0.919		*p* = 0.115		*p* = 0.639		*p* = 0.412	

MPS, mucopolysaccharidosis; ERT, enzyme replacement therapy; MS, mitral stenosis; MR, mitral regurgitation; AS, aortic stenosis; AR, aortic regurgitation

## Discussion

To the best of our knowledge, this is the first report to delineate the natural progression of cardiac features and long-term effects of ERT in a single Asian population with MPS II and compare the results with normal values, including young adults, based on the report of Kampmann et al. [43]. Our results demonstrated the high prevalence of cardiac hypertrophy, aortic dilatation, increased thickness of the IVS, normal systolic function, and valvular heart disease in MPS II patients. There is no difference in cardiac findings between severe and mild forms of MPS II. The existence and severity of cardiac hypertrophy and valvular heart disease in these patients worsened with increasing age, reinforcing the concept of the progressive nature of this disease. ERT for MPS II appeared to be effective in stabilizing or reducing cardiac hypertrophy, however, the effects on valvulopathy were limited. Our results are consistent with those of previous studies [6–8, 10–13, 15, 26, 28–30].

Most of the MPS II patients in this cohort (85%) had cardiac abnormalities. Only seven patients who were referred from newborn screening programs (patients No. 1, 2, 4–6, 12, 23) had normal cardiac features by echocardiography. Echocardiographic assessments revealed mean *z*-scores of LVMI, IVSd, LVPWd, and aortic diameter of 1.05, 2.66, 0.86 and 1.97, respectively. *Z*-scores > 2 were identified in 31%, 54%, 13%, and 46% of the LVMI, IVSd, LVPWd, and aortic diameter values, respectively. Although LV systolic function was abnormal in only one patient (2%), diastolic dysfunction with a reversed E/A ratio (< 1) was identified in eight patients (17%). We are among the first to present E/A ratio of MPS patients. The Hunter Outcome Survey (HOS) data reported by Kampmann et al. [44] showed that LV hypertrophy was present in 48% (24/50) of their MPS II patients. Despite the high prevalence of valvular dysfunction in their cohort, few patients had reductions in ejection fraction. Leal et al. [12] reported that LV hypertrophy and diastolic dysfunction emerged at an early stage, however, LV dilatation and systolic dysfunction occurred at an older age and later disease stage, which is consistent with our results.

Bolourchi et al. [45] reported a high prevalence [35% (12/34)] of aortic root dilatation in their cohort of MPS I–VII patients. Similarly, 46% (22/48) of our MPS II patients had aortic root dilatation. Thus routine screening for this potentially crucial factor should be incorporated into the multidisciplinary care for these patients.

Deformed mitral or aortic valves with varying degrees of severity were commonly found (77%) in our patients. Similarly, the HOS data reported by Kampmann et al. [44] showed that valvular heart disease was present in 63% (63/100) of their MPS II patients. In our study, mitral valve abnormalities (65%) were more prevalent than aortic valve abnormalities (52%), and valvular regurgitation (65%) was more common than valvular stenosis (35%), which is consistent with previous studies [6, 7, 10–12]. In addition, the most prevalent cardiac valve abnormality was MR (54%), followed by AR (35%), MS (31%), and AS (27%). In agreement with the report by Mohan et al. [8], valvular stenosis and regurgitation in our cohort worsened with increasing age, which is consistent with the progressive nature of this disease.

Only a few studies have reported the LV remodeling pattern in patients with MPS [17]. Each of these three patterns was associated with a higher risk of subsequent cardiovascular events with the composite of cardiovascular death, myocardial infarction, stroke, heart failure, or resuscitated cardiac arrest, and a progressively worse prognosis than a normal LV morphology.

Previous studies have reported that cardiac disease can occur insidiously and lead to early mortality in patients with MPS II [20, 21, 23]. Echocardiography is an important diagnostic technique to assess cardiac valves, ventricular dimensions and function [11]. Therefore, comprehensive physical examinations and echocardiography should be conducted when MPS is diagnosed, followed by regular cardiac function follow-up examinations [3]. Without routine cardiac monitoring, cardiac lesions may remain undetected due to insufficient physical activity caused by pulmonary function impairment and skeletal dysplasia.

In our cohort, natural deterioration of hypertrophic cardiomyopathy and valvulopathy were noted after 2.6–17.0 years of follow-up echocardiography in 12 patients who did not receive ERT or HSCT compared to baseline data. However, the *z*-scores of LVMI, IVSd and LVPWd in nine patients who received ERT for 1.0–12.4 years all revealed stabilization of the natural progression of hypertrophic cardiomyopathy. The ages of the patients who received ERT are much older than those who did not receive ERT. The patients who received ERT started with higher *z*-scores. Despite this older age, the changes over time were smaller than those experienced by the young patients who did not receive ERT. Therefore, stabilization could be considered a positive response to treatment in a progressive disease such as MPS II. This suggests that ERT has some effect on GAG accumulation in the cardiac tissue of patients with MPS, and that this is effective in alleviating the progression of cardiac hypertrophy. However, ERT seemed to have little or no effect on valvulopathy, which is consistent with previous studies [10, 13, 15, 19, 28, 46–48]. Braunlin et al. [47] and Kampmann et al. [48] reported that ERT may have better long-term results when started at an early age for patients with MPS VI, which is in agreement with our results. Several sibling control studies have also reported that ERT may prevent or delay the development of valvular heart disease when started early in life [49–53]. In the recent decade, the increasing clinical awareness of MPS disease and increased ability to make a confirmative diagnosis has made an earlier diagnosis possible. Due to the progressive nature of MPS, initiating ERT before the occurrence of irreversible cardiac damage may contribute to a better clinical outcome. As a result, making an early diagnosis through newborn screening programs or high-risk population screening programs is very important [54–57].

### Limitations

As a retrospective and uncontrolled study there was no healthy control group, so we could not compare the echocardiographic parameters between the patients and healthy controls. In addition, some patients in this cohort did not have follow-up echocardiographic data to compare with the baseline data. We used reference values from a Caucasian population due to the lack of reference values from an Asian population. The small number of patients with MPS II reflects the rare nature of this genetic disorder. Moreover, both the age range (0.1–27.9 years) and degree of disease severity varied considerably. Consequently, studies with larger cohorts and longer follow-up periods are required.

##  Conclusion

High prevalence rates of cardiac hypertrophy, aortic dilatation, and valvular heart disease were observed in the Taiwanese patients with MPS II in this study. The cardiac abnormalities in these patients worsened with increasing age, reinforcing the concept of the progressive nature of this disease. ERT appeared to be effective in stabilizing or reducing cardiac hypertrophy, however, there was limited effect on valvulopathy. Therefore, it is very important to make an early diagnosis through newborn screening programs or high-risk population screening programs in order to initiate ERT before the occurrence of irreversible cardiac damage. These findings and the follow-up data can be used to develop quality of care strategies for these patients.

## Supplementary Information

**Additional file 1: Tables 1 and 2**. The detailed data of 12 patients with MPS II who had echocardiographic examinations after 2.6–17.0 years of follow-up and had not received ERT or HSCT. **Tables 3 and 4**. The baseline and follow-up echocardiographic parameters of nine patients with MPS II who received ERT for 1.0–12.4 years.

## Data Availability

Not applicable. There are no other supporting data and materials since all of them are in this article.

## Abbreviations

MPS: Mucopolysaccharidosis
GAGs: Glycosaminoglycans
IDS: Iduronate-2-sulfatase
ERT: Enzyme replacement therapy
LVMI: Left ventricular mass index
HSCT: Hematopoietic stem cell transplantation
LV: Left ventricular
E/A: Ratio between early and late (atrial) ventricular filling velocity
AS: Aortic stenosis
MS: Mitral stenosis
RVDd: Right ventricular end-diastolic dimension
IVSd: Interventricular septal end-diastolic dimension
LVIDd: Left ventricular end-diastolic dimension
LVIDs: Left ventricular end-systolic dimension
LVPWd: Left ventricular posterior wall end-diastolic dimension
LAD: Left atrial dimension
RWT: Relative wall thickness
MR: Mitral regurgitation
AR: Aortic regurgitation

## References

[1] Neufeld EF, Muenzer J, Scriver CR, Beaudet AL, Sly WS, Valle D, Childs B, Kinzler KW, Vogelstein B (2001). The mucoplysaccharidoses. The metabolic and molecular bases of inherited disease.

[2] Lin HY, Lee CL, Lo YT, Wang TJ, Huang SF, Chen TL, Wang YS, Niu DM, Chuang CK, Lin SP (2018). The relationships between urinary glycosaminoglycan levels and phenotypes of mucopolysaccharidoses. Mol Genet Genomic Med.

[3] Scarpa M, Almássy Z, Beck M, Bodamer O, Bruce IA, De Meirleir L (2011). Mucopolysaccharidosis type II: European recommendations for the diagnosis and multidisciplinary management of a rare disease. Orphanet J Rare Dis.

[4] Khan SA, Peracha H, Ballhausen D, Wiesbauer A, Rohrbach M, Gautschi M (2017). Epidemiology of mucopolysaccharidoses. Mol Genet Metab.

[5] Lin HY, Lin SP, Chuang CK, Niu DM, Chen MR, Tsai FJ (2009). Incidence of the mucopolysaccharidoses in Taiwan, 1984–2004. Am J Med Genet A.

[6] Wippermann CF, Beck M, Schranz D, Huth R, Michel-Behnke I, Jüngst BK (1995). Mitral and aortic regurgitation in 84 patients with mucopolysaccharidoses. Eur J Pediatr.

[7] Dangel JH (1998). Cardiovascular changes in children with mucopolysaccharide storage diseases and related disorders–clinical and echocardiographic findings in 64 patients. Eur J Pediatr.

[8] Mohan UR, Hay AA, Cleary MA, Wraith JE, Patel RG (2002). Cardiovascular changes in children with mucopolysaccharide disorders. Acta Paediatr.

[9] Chen MR, Lin SP, Hwang HK, Yu CH (2005). Cardiovascular changes in mucopolysaccharidoses in Taiwan. Acta Cardiol.

[10] Fesslová V, Corti P, Sersale G, Rovelli A, Russo P, Mannarino S (2009). The natural course and the impact of therapies of cardiac involvement in the mucopolysaccharidoses. Cardiol Young.

[11] Braunlin EA, Harmatz PR, Scarpa M, Furlanetto B, Kampmann C, Loehr JP (2011). Cardiac disease in patients with mucopolysaccharidosis: presentation, diagnosis and management. J Inherit Metab Dis.

[12] Leal GN, de Paula AC, Leone C, Kim CA (2010). Echocardiographic study of paediatric patients with mucopolysaccharidosis. Cardiol Young.

[13] Brands MM, Frohn-Mulder IM, Hagemans ML, Hop WC, Oussoren E, Helbing WA (2013). Mucopolysaccharidosis: cardiologic features and effects of enzyme-replacement therapy in 24 children with MPS I, II and VI. J Inherit Metab Dis.

[14] Lin SM, Lin HY, Chuang CK, Lin SP, Chen MR (2014). Cardiovascular abnormalities in Taiwanese patients with mucopolysaccharidosis. Mol Genet Metab.

[15] Lin HY, Chuang CK, Chen MR, Lin SM, Hung CL, Chang CY (2016). Cardiac structure and function and effects of enzyme replacement therapy in patients with mucopolysaccharidoses I, II, IVA and VI. Mol Genet Metab.

[16] Braunlin E, Wang R (2016). Cardiac issues in adults with the mucopolysaccharidoses: current knowledge and emerging needs. Heart.

[17] Lin HY, Chuang CK, Lee CL, Chen MR, Sung KT, Lin SM (2020). Cardiac evaluation using two-dimensional speckle-tracking echocardiography and conventional echocardiography in Taiwanese patients with mucopolysaccharidoses. Diagnostics.

[18] Lin HY, Chen MR, Lee CL, Lin SM, Hung CL, Niu DM (2021). Aortic root dilatation in Taiwanese patients with mucopolysaccharidoses and the long-term effects of enzyme replacement therapy. Diagnostics (Basel).

[19] Braunlin EA, Berry JM, Whitley CB (2006). Cardiac findings after enzyme replacement therapy for mucopolysaccharidosis type I. Am J Cardiol.

[20] Lin HY, Lin SP, Chuang CK, Chen MR, Chen BF, Wraith JE (2005). Mucopolysaccharidosis I under enzyme replacement therapy with laronidase—a mortality case with autopsy report. J Inherit Metab Dis.

[21] Jones SA, Almássy Z, Beck M, Burt K, Clarke JT, Giugliani R (2009). Mortality and cause of death in mucopolysaccharidosis type II-a historical review based on data from the Hunter Outcome Survey (HOS). J Inherit Metab Dis.

[22] Lavery C, Hendriksz C (2015). Mortality in patients with Morquio syndrome A. JIMD Rep.

[23] Lin HY, Chuang CK, Huang YH, Tu RY, Lin FJ, Lin SJ (2016). Causes of death and clinical characteristics of 34 patients with mucopolysaccharidosis II in Taiwan from 1995–2012. Orphanet J Rare Dis.

[24] Muenzer J, Wraith JE, Beck M, Giugliani R, Harmatz P, Eng CM (2006). A phase II/III clinical study of enzyme replacement therapy with idursulfase in mucopolysaccharidosis II (Hunter syndrome). Genet Med.

[25] Wraith JE, Scarpa M, Beck M, Bodamer OA, De Meirleir L, Guffon N (2008). Mucopolysaccharidosis type II (Hunter syndrome): a clinical review and recommendations for treatment in the era of enzyme replacement therapy. Eur J Pediatr.

[26] Okuyama T, Tanaka A, Suzuki Y, Ida H, Tanaka T, Cox GF (2010). Japan Elaprase Treatment (JET) study: idursulfase enzyme replacement therapy in adult patients with attenuated Hunter syndrome (Mucopolysaccharidosis II, MPS II). Mol Genet Metab.

[27] Tajima G, Sakura N, Kosuga M, Okuyama T, Kobayashi M (2013). Effects of idursulfase enzyme replacement therapy for mucopolysaccharidosis type II when started in early infancy: comparison in two siblings. Mol Genet Metab.

[28] Parini R, Rigoldi M, Tedesco L, Boffi L, Brambilla A, Bertoletti S (2015). Enzymatic replacement therapy for Hunter disease: up to 9 years experience with 17 patients. Mol Genet Metab Rep.

[29] Lampe C, Bosserhoff AK, Burton BK, Giugliani R, de Souza CF, Bittar C (2014). Long-term experience with enzyme replacement therapy (ERT) in MPS II patients with a severe phenotype: an international case series. J Inherit Metab Dis.

[30] Bilginer Gurbuz B, Aypar E, Coskun T, Alehan D, Dursun A, Tokatli A (2019). The effectiveness of enzyme replacement therapy on cardiac findings in patients with mucopolysaccharidosis. J Pediatr Endocrinol Metab.

[31] Chuang CK, Lin HY, Wang TJ, Tsai CC, Liu HL, Lin SP (2014). A modified liquid chromatography/tandem mass spectrometry method for predominant disaccharide units of urinary glycosaminoglycans in patients with mucopolysaccharidoses. Orphanet J Rare Dis.

[32] Lin HY, Lo YT, Wang TJ, Huang SF, Tu RY, Chen TL (2019). Normalization of glycosaminoglycan-derived disaccharides detected by tandem mass spectrometry assay for the diagnosis of mucopolysaccharidosis. Sci Rep.

[33] Lin HY, Tu RY, Chern SR, Lo YT, Fran S, Wei FJ (2020). Identification and functional characterization of IDS gene mutations underlying Taiwanese Hunter Syndrome (mucopolysaccharidosis type II). Int J Mol Sci.

[34] Lang RM, Badano LP, Mor-Avi V, Afilalo J, Armstrong A, Ernande L (2015). Recommendations for cardiac chamber quantification by echocardiography in adults: an update from the American Society of Echocardiography and the European Association of Cardiovascular Imaging. Eur Heart J Cardiovasc Imaging.

[35] Einarsen E, Cramariuc D, Lønnebakken MT, Boman K, Gohlke-Bärwolf C, Chambers JB (2017). Comparison of frequency of ischemic cardiovascular events in patients with aortic stenosis with versus without asymmetric septal hypertrophy (from the SEAS Trial). Am J Cardiol.

[36] Eidem BW, McMahon CJ, Cohen RR, Wu J, Finkelshteyn I, Kovalchin JP (2004). Impact of cardiac growth on Doppler tissue imaging velocities: a study in healthy children. J Am Soc Echocardiogr.

[37] Baumgartner H, Hung J, Bermejo J, Chambers JB, Evangelista A, Griffin BP (2009). Echocardiographic assessment of valve stenosis: EAE/ASE recommendations for clinical practice. J Am Soc Echocardiogr.

[38] Lancellotti P, Tribouilloy C, Hagendorff A, Popescu BA, Edvardsen T, Pierard LA (2013). Recommendations for the echocardiographic assessment of native valvular regurgitation: an executive summary from the European Association of Cardiovascular Imaging. Eur Heart J Cardiovasc Imaging.

[39] Lin HY, Chen MR, Lin SM, Hung CL, Niu DM, Chuang CK (2018). Cardiac features and effects of enzyme replacement therapy in Taiwanese patients with mucopolysaccharidosis IVA. Orphanet J Rare Dis.

[40] Lin HY, Chen MR, Lin SM, Hung CL, Niu DM, Chang TM (2019). Cardiac characteristics and natural progression in taiwanese patients with mucopolysaccharidosis III. Orphanet J Rare Dis.

[41] Lang RM, Bierig M, Devereux RB, Flachskampf FA, Foster E, Pellikka PA (2005). Recommendations for chamber quantification: a report from the American Society of Echocardiography's Guidelines and Standards Committee and the Chamber Quantification Writing Group, developed in conjunction with the European Association of Echocardiography, a branch of the European Society of Cardiology. J Am Soc Echocardiogr.

[42] Foster BJ, Mackie AS, Mitsnefes M, Ali H, Mamber S, Colan SD (2008). A novel method of expressing left ventricular mass relative to body size in children. Circulation.

[43] Kampmann C, Wiethoff CM, Wenzel A, Stolz G, Betancor M, Wippermann CF (2000). Normal values of M mode echocardiographic measurements of more than 2000 healthy infants and children in central Europe. Heart.

[44] Kampmann C, Beck M, Morin I, Loehr JP (2011). Prevalence and characterization of cardiac involvement in Hunter syndrome. J Pediatr.

[45] Bolourchi M, Renella P, Wang RY (2016). Aortic root dilatation in mucopolysaccharidosis I–VII. Int J Mol Sci.

[46] Wraith JE, Beck M, Lane R, van der Ploeg A, Shapiro E, Xue Y (2007). Enzyme replacement therapy in patients who have mucopolysaccharidosis I and are younger than 5 years: results of a multinational study of recombinant human alpha-L-iduronidase (laronidase). Pediatrics.

[47] Braunlin E, Rosenfeld H, Kampmann C, Johnson J, Beck M, Giugliani R (2013). Enzyme replacement therapy for mucopolysaccharidosis VI: long-term cardiac effects of galsulfase (Naglazyme®) therapy. J Inherit Metab Dis.

[48] Kampmann C, Lampe C, Whybra-Trümpler C, Wiethoff CM, Mengel E, Arash L (2014). Mucopolysaccharidosis VI: cardiac involvement and the impact of enzyme replacement therapy. J Inherit Metab Dis.

[49] Gabrielli O, Clarke LA, Bruni S, Coppa GV (2010). Enzyme-replacement therapy in a 5-month-old boy with attenuated presymptomatic MPS I: 5-year follow-up. Pediatrics.

[50] McGill JJ, Inwood AC, Coman DJ, Lipke ML, de Lore D, Swiedler SJ (2010). Enzyme replacement therapy for mucopolysaccharidosis VI from 8 weeks of age—a sibling control study. Clin Genet.

[51] Al-Sannaa NA, Bay L, Barbouth DS, Benhayoun Y, Goizet C, Guelbert N (2015). Early treatment with laronidase improves clinical outcomes in patients with attenuated MPS I: a retrospective case series analysis of nine sibships. Orphanet J Rare Dis.

[52] Franco JF, Soares DC, Torres LC, Leal GN, Cunha MT, Honjo RS, et al: Short Communication Impact of early enzyme-replacement therapy for mucopolysaccharidosis VI: results of a long-term follow-up of Brazilian siblings. Genet Mol Res 2016, 15(1). 10.4238/gmr.15017850 26910003

[53] Furujo M, Kosuga M, Okuyama T (2017). Enzyme replacement therapy attenuates disease progression in two Japanese siblings with mucopolysaccharidosis type VI: 10-year follow up. Mol Genet Metab Rep.

[54] Chuang CK, Lin HY, Wang TJ, Huang YH, Chan MJ, Liao HC (2018). Status of newborn screening and follow up investigations for mucopolysaccharidoses I and II in Taiwan. Orphanet J Rare Dis.

[55] Chan MJ, Liao HC, Gelb MH, Chuang CK, Liu MY, Chen HJ (2019). Taiwan national newborn screening program by tandem mass spectrometry for mucopolysaccharidoses types I, II, and VI. J Pediatr.

[56] Lin SP, Lin HY, Wang TJ, Chang CY, Lin CH, Huang SF (2013). A pilot newborn screening program for mucopolysaccharidosis type I in Taiwan. Orphanet J Rare Dis.

[57] Lin HY, Lee CL, Lo YT, Tu RY, Chang YH, Chang CY (2019). An at-risk population screening program for mucopolysaccharidoses by measuring urinary glycosaminoglycans in Taiwan. Diagnostics (Basel).

