# Supplementary material for: Natural progression of cardiac features and long-term effects of enzyme replacement therapy in Taiwanese patients with mucopolysaccharidosis II
Source: Orphanet J Rare Dis. 2021 Feb 23;16:99. doi: 10.1186/s13023-021-01743-2 (PMC7903682; doi:10.1186/s13023-021-01743-2)
Supplement: Supplementary file 1 — Additional file 1: Tables 1 and 2. The detailed data of 12 patients with MPS II who had echocardiographic examinations after 2.6–17.0 years of follow-up and had not received ERT or HSCT. Tables 3 and 4. The baseline and follow-up echocardiographic parameters of nine patients with MPS II who received ERT for 1.0–12.4 years. [file 13023_2021_1743_MOESM1_ESM.doc]

**Supplement Table 1.** Twelve patients with MPS II who had echocardiographic examinations after 2.6-17.0 years of follow-up without ERT compared with the baseline data.

| No. | MPS type | Gender | Age at baseline (years) | Age at follow-up (years) | Duration (years) | LVMI (z score) | | Change  (z score) | IVSd (z score) | | Change  (z score) | LVPWd (z score) | | Change  (z score) |
| --- | --- | --- | --- | --- | --- | --- | --- | --- | --- | --- | --- | --- | --- | --- |
|  |  |  |  |  |  | Baseline | Follow-up |  | Baseline | Follow-up |  | Baseline | Follow-up |  |
| 7 | II (S) | M | 2.1 | 6.7 | 4.6 | 2.49 | 5.17 | 2.68 | 1.73 | 1.43 | -0.31 | 1.09 | 3.63 | 2.54 |
| 14 | II (M) | M | 4.5 | 15.4 | 10.9 | 1.87 | 2.95 | 1.08 | 1.01 | 2.22 | 1.21 | 0.66 | 0.68 | 0.02 |
| 16 | II (M) | M | 4.7 | 18.7 | 14.0 | 1.96 | 4.25 | 2.29 | 3.04 | 2.70 | -0.34 | 0.63 | 2.77 | 2.14 |
| 18 | II (S) | M | 5.0 | 11.6 | 6.6 | 0.60 | 2.15 | 1.55 | 2.07 | 2.26 | 0.19 | 0.57 | 1.95 | 1.38 |
| 19 | II (S) | M | 5.1 | 8.0 | 2.9 | 1.57 | 1.43 | -0.14 | 4.61 | 4.13 | -0.48 | 1.57 | 0.80 | -0.77 |
| 20 | II (M) | M | 5.6 | 13.2 | 7.6 | -0.43 | 2.75 | 3.18 | 1.45 | 2.31 | 0.86 | -1.80 | 2.13 | 3.93 |
| 21 | II (M) | M | 5.6 | 17.5 | 11.9 | -1.21 | 1.29 | 2.50 | 1.13 | 4.56 | 3.43 | 0.38 | 0.56 | 0.18 |
| 24 | II (M) | M | 6.9 | 10.8 | 3.9 | 0.03 | -0.56 | -0.59 | 1.83 | 2.97 | 1.13 | 0.53 | 1.57 | 1.05 |
| 26 | II (S) | M | 7.1 | 17.7 | 10.6 | -1.46 | 0.66 | 2.12 | 2.83 | 3.16 | 0.33 | 0.70 | 0.65 | -0.06 |
| 30 | II (S) | M | 9.9 | 12.5 | 2.6 | 1.77 | 1.29 | -0.48 | 2.50 | 5.20 | 2.70 | -0.18 | 0.36 | 0.55 |
| 37 | II (S) | M | 12.1 | 29.0 | 17.0 | -0.44 | 2.89 | 3.33 | 0.62 | 10.11 | 9.49 | 1.18 | 0.57 | -0.61 |
| 45 | II (M) | M | 19.1 | 24.1 | 5.0 | 3.19 | 3.96 | 0.77 | 6.00 | 9.33 | 3.33 | 1.57 | 6.18 | 4.61 |
| Mean | | | 7.3 | 15.4 | 8.1 | 0.83 | 2.35 | 1.52 | 2.40 | 4.20 | 1.80 | 0.58 | 1.82 | 1.25 |
| *p* value | | | | | | *p*=0.027 | |  | *p*=0.065 | |  | *p*=0.036 | |  |
| MPS, mucopolysaccharidosis; ERT, enzyme replacement therapy; LVMI, left ventricular mass index; IVSd, interventricular septal end-diastolic dimension; LVPWd, left ventricular posterior wall end-diastolic dimension; MPS II (S), severe form; MPS II (M), mild form. | | | | | | | | | | | | | | |

**Supplement Table 2.** Twelve patients with MPS II who had echocardiographic examinations after 2.6-17.0 years of follow-up without ERT compared with the baseline data.

| No. | MPS type | Gender | Age at baseline (years) | Age at follow-up (years) | Duration (years) | Severity score of MS | | Change of severity score | Severity score of MR | | Change of severity score | Severity score of AS | | Change of severity score | Severity score of AR | | Change of severity score |
| --- | --- | --- | --- | --- | --- | --- | --- | --- | --- | --- | --- | --- | --- | --- | --- | --- | --- |
|  |  |  |  |  |  | Baseline | Follow-up |  | Baseline | Follow-up |  | Baseline | Follow-up |  | Baseline | Follow-up |  |
| 7 | II (S) | M | 2.1 | 6.7 | 4.6 | 0.0 | 0.0 | 0.0 | 1.5 | 2.0 | 0.5 | 0.0 | 0.0 | 0.0 | 0.0 | 0.0 | 0.0 |
| 14 | II (M) | M | 4.5 | 15.4 | 10.9 | 0.0 | 0.0 | 0.0 | 2.0 | 2.0 | 0.0 | 0.0 | 0.0 | 0.0 | 1.5 | 1.5 | 0.0 |
| 16 | II (M) | M | 4.7 | 18.7 | 14.0 | 0.0 | 2.0 | 2.0 | 0.0 | 0.5 | 0.5 | 0.0 | 2.0 | 2.0 | 0.0 | 2.0 | 2.0 |
| 18 | II (S) | M | 5.0 | 11.6 | 6.6 | 0.0 | 0.0 | 0.0 | 1.0 | 1.0 | 0.0 | 0.0 | 1.0 | 1.0 | 0.0 | 0.0 | 0.0 |
| 19 | II (S) | M | 5.1 | 8.0 | 2.9 | 1.0 | 1.0 | 0.0 | 0.0 | 1.0 | 1.0 | 1.0 | 1.0 | 0.0 | 0.0 | 0.0 | 0.0 |
| 20 | II (M) | M | 5.6 | 13.2 | 7.6 | 0.0 | 1.0 | 1.0 | 1.0 | 1.0 | 0.0 | 0.0 | 0.0 | 0.0 | 0.0 | 1.0 | 1.0 |
| 21 | II (M) | M | 5.6 | 17.5 | 11.9 | 0.0 | 0.0 | 0.0 | 0.0 | 0.0 | 0.0 | 0.0 | 1.0 | 1.0 | 0.0 | 2.0 | 2.0 |
| 24 | II (M) | M | 6.9 | 10.8 | 3.9 | 0.0 | 0.0 | 0.0 | 0.0 | 1.0 | 1.0 | 0.0 | 0.0 | 0.0 | 0.0 | 0.0 | 0.0 |
| 26 | II (S) | M | 7.1 | 17.7 | 10.6 | 0.0 | 1.0 | 1.0 | 0.0 | 1.0 | 1.0 | 0.0 | 1.0 | 1.0 | 0.0 | 0.0 | 0.0 |
| 30 | II (S) | M | 9.9 | 12.5 | 2.6 | 1.0 | 1.0 | 0.0 | 0.0 | 1.0 | 1.0 | 1.0 | 1.0 | 0.0 | 0.0 | 1.0 | 1.0 |
| 37 | II (S) | M | 12.1 | 29.0 | 17.0 | 1.0 | 1.0 | 0.0 | 1.0 | 2.5 | 1.5 | 0.0 | 1.0 | 1.0 | 0.0 | 3.0 | 3.0 |
| 45 | II (M) | M | 19.1 | 24.1 | 5.0 | 1.0 | 2.0 | 1.0 | 1.0 | 1.0 | 0.0 | 1.0 | 1.5 | 0.5 | 0.0 | 0.0 | 0.0 |
| Mean | | | 7.3 | 15.4 | 8.1 | 0.33 | 0.75 | 0.42 | 0.63 | 1.17 | 0.54 | 0.25 | 0.79 | 0.54 | 0.13 | 0.88 | 0.75 |
| *p* value | | | | | | *p*=0.123 | |  | *p*=0.071 | |  | *p*=0.028 | |  | *p*=0.032 | |  |
| MPS, mucopolysaccharidosis; ERT, enzyme replacement therapy; MS, mitral stenosis; MR, mitral regurgitation; AS, aortic stenosis; AR, aortic regurgitation. Severity of valvular stenosis and regurgitation (MS, MR, AS, AR) were estimated and graded on the following scores: 0 (none), 1 (mild), 2 (moderate), and 3 (severe); MPS II (S), severe form; MPS II (M), mild form. | | | | | | | | | | | | | | | | | |

**Supplement Table 3.** Baseline and follow-up echocardiographic parameters of nine patients with MPS II who received ERT for 1.0-12.4 years.

| No. | MPS type | Gender | Age at baseline (years) | Age at follow-up (years) | ERT duration (years) | LVMI (z score) | | Change  (z score) | IVSd (z score) | | Change  (z score) | LVPWd (z score) | | Change  (z score) |
| --- | --- | --- | --- | --- | --- | --- | --- | --- | --- | --- | --- | --- | --- | --- |
|  |  |  |  |  |  | Baseline | Follow-up |  | Baseline | Follow-up |  | Baseline | Follow-up |  |
| 25 | II (M) | M | 7.0 | 15.4 | 8.4 | 4.03 | 3.39 | -0.64 | 1.47 | 2.31 | 0.84 | 1.57 | 3.08 | 1.51 |
| 33 | II (M) | M | 10.9 | 11.9 | 1.0 | 2.16 | 1.58 | -0.58 | 2.88 | 0.88 | -1.99 | 1.42 | 0.85 | -0.57 |
| 20 | II (M) | M | 13.2 | 25.7 | 12.4 | 2.75 | 1.54 | -1.21 | 2.31 | 1.69 | -0.61 | 2.13 | 0.17 | -1.96 |
| 42 | II (M) | M | 14.8 | 23.3 | 8.5 | 2.81 | 2.90 | 0.09 | 2.94 | 2.55 | -0.40 | 2.35 | 2.07 | -0.28 |
| 14 | II (M) | M | 15.4 | 17.7 | 2.2 | 2.95 | 4.33 | 1.38 | 2.22 | 4.74 | 2.52 | 0.68 | 3.17 | 2.48 |
| 21 | II (M) | M | 17.5 | 29.8 | 12.3 | 1.29 | 2.81 | 1.52 | 4.56 | 1.18 | -3.37 | 0.56 | 2.36 | 1.80 |
| 44 | II (M) | M | 18.1 | 26.6 | 8.6 | 3.37 | 3.73 | 0.36 | 4.82 | 4.67 | -0.15 | 1.04 | 1.67 | 0.63 |
| 16 | II (M) | M | 18.7 | 21.5 | 2.7 | 4.25 | 3.64 | -0.61 | 2.7 | 3.34 | 0.64 | 2.77 | 0.98 | -1.78 |
| 46 | II (M) | M | 21.2 | 29.6 | 8.4 | 1.44 | 1.54 | 0.10 | 3.18 | 3.59 | 0.41 | 2.55 | 5.38 | 2.83 |
| Mean | | | 15.2 | 22.4 | 7.2 | 2.78 | 2.83 | 0.05 | 3.01 | 2.77 | -0.24 | 1.68 | 2.19 | 0.52 |
| *p* value | | | | | | *p*=0.927 | |  | *p*=0.697 | |  | *p*=0.393 | |  |
| MPS, mucopolysaccharidosis; ERT, enzyme replacement therapy; LVMI, left ventricular mass index; IVSd, interventricular septal end-diastolic dimension; LVPWd, left ventricular posterior wall end-diastolic dimension; MPS II (S), severe form; MPS II (M), mild form. | | | | | | | | | | | | | | |

**Supplement Table 4.** Baseline and follow-up echocardiographic parameters of nine patients with MPS II who received ERT for 1.0-12.4 years.

| No. | MPS type | Gender | Age at baseline (years) | Age at follow-up (years) | ERT duration (years) | Severity score of MS | | Change of severity score | Severity score of MR | | Change of severity score | Severity score of AS | | Change of severity score | Severity score of AR | | Change of severity score |
| --- | --- | --- | --- | --- | --- | --- | --- | --- | --- | --- | --- | --- | --- | --- | --- | --- | --- |
|  |  |  |  |  |  | Baseline | Follow-up |  | Baseline | Follow-up |  | Baseline | Follow-up |  | Baseline | Follow-up |  |
| 25 | II (M) | M | 7.0 | 15.4 | 8.4 | 0.0 | 1.0 | 1.0 | 0.0 | 0.5 | 0.5 | 0.0 | 1.0 | 1.0 | 1.0 | 2.5 | 1.5 |
| 33 | II (M) | M | 10.9 | 11.9 | 1.0 | 0.0 | 0.0 | 0.0 | 1.0 | 0.0 | -1.0 | 0.0 | 1.0 | 1.0 | 2.0 | 2.0 | 0.0 |
| 20 | II (M) | M | 13.2 | 25.7 | 12.4 | 1.0 | 1.0 | 0.0 | 1.0 | 1.5 | 0.5 | 0.0 | 1.0 | 1.0 | 1.0 | 1.5 | 0.5 |
| 42 | II (M) | M | 14.8 | 23.3 | 8.5 | 0.0 | 1.0 | 1.0 | 1.0 | 1.0 | 0.0 | 0.0 | 1.0 | 1.0 | 1.5 | 3.0 | 1.5 |
| 14 | II (M) | M | 15.4 | 17.7 | 2.2 | 0.0 | 0.0 | 0.0 | 2.0 | 1.0 | -1.0 | 0.0 | 0.0 | 0.0 | 1.5 | 1.0 | -0.5 |
| 21 | II (M) | M | 17.5 | 29.8 | 12.3 | 0.0 | 1.0 | 1.0 | 0.0 | 1.5 | 1.5 | 1.0 | 2.0 | 1.0 | 2.0 | 2.5 | 0.5 |
| 44 | II (M) | M | 18.1 | 26.6 | 8.6 | 0.0 | 1.0 | 1.0 | 1.5 | 2.0 | 0.5 | 0.0 | 1.0 | 1.0 | 1.0 | 2.0 | 1.0 |
| 16 | II (M) | M | 18.7 | 21.5 | 2.7 | 2.0 | 2.0 | 0.0 | 0.5 | 0.0 | -0.5 | 2.0 | 2.0 | 0.0 | 2.0 | 2.0 | 0.0 |
| 46 | II (M) | M | 21.2 | 29.6 | 8.4 | 1.0 | 1.0 | 0.0 | 0.0 | 0.0 | 0.0 | 1.0 | 1.0 | 0.0 | 1.0 | 0.0 | -1.0 |
| Mean | | | 15.2 | 22.4 | 7.2 | 0.44 | 0.88 | 0.44 | 0.78 | 0.75 | 0.06 | 0.44 | 1.13 | 0.67 | 1.44 | 1.88 | 0.39 |
| *p* value | | | | | | *p*=0.176 | |  | *p*=0.874 | |  | *p*=0.050 | |  | *p*=0.267 | |  |
| MPS, mucopolysaccharidosis; ERT, enzyme replacement therapy; MS, mitral stenosis; MR, mitral regurgitation; AS, aortic stenosis; AR, aortic regurgitation. Severity of valvular stenosis and regurgitation (MS, MR, AS, AR) were estimated and graded on the following scores: 0 (none), 1 (mild), 2 (moderate), and 3 (severe); MPS II (S), severe form; MPS II (M), mild form. | | | | | | | | | | | | | | | | | |
